# Successful Treatment of Iatrogenic Vertebral Pseudoaneurysm Using Pipeline Embolization Device

**DOI:** 10.1155/2014/341748

**Published:** 2014-09-03

**Authors:** Sudheer Ambekar, Mayur Sharma, Donald Smith, Hugo Cuellar

**Affiliations:** ^1^Department of Neurosurgery, Louisiana State University Health Sciences Center, 1501 Kings Highway, Shreveport, LA 71103, USA; ^2^Center of Neuromodulation, Wexner Medical Center, The Ohio State University, Columbus, OH 43210, USA

## Abstract

Traumatic pseudoaneurysms are uncommon and one of the most difficult lesions to treat. Traditional treatment methods have focused on parent vessel sacrifice with or without revascularization. We report the case of a patient who underwent successful treatment of an iatrogenic extracranial vertebral artery pseudoaneurysm using the Pipeline Embolization Device. A 47-year-old man sustained an inadvertent injury to the left vertebral artery during C1-C2 fixation. Subsequent imaging revealed an iatrogenic vertebral artery pseudoaneurysm. Immediate angiogram was normal. A repeat angiogram done after 3 days of the surgery revealed a vertebral artery pseudoaneurysm. He underwent aneurysm exclusion and vascular reconstruction using the Pipeline Embolization Device. Although flow-diverting stents are currently not being used for treating traumatic pseudoaneurysms, their use may be considered in such cases if active bleeding has ceased. In our case, the patient did well and the aneurysm was excluded from circulation while reconstructing the vessel wall.

## 1. Introduction

Vertebral artery pseudoaneurysms may arise due to penetrating or blunt trauma, arterial dissection, associated collagen vascular disease, or following surgery. The extracranial segment (V3) of the vertebral artery is the most vulnerable to iatrogenic injury due to its course outside the transverse foramen and close proximity to C1. The incidence of vertebral artery (VA) injury during craniocervical fusion surgery has been reported between 0% and 5.8% [1, 2]. VA injury during these procedures may lead to massive hemorrhage, arterial infarction, and, sometimes, death. In a few cases, delayed formation of pseudoaneurysm may be observed. Although spontaneous resolution of these pseudoaneurysms has been reported, rupture is observed in about 31% to 54% of patients [3]. Therefore, prompt identification and management of these lesions are paramount to successful outcome.

Various treatment strategies for treatment of iatrogenic pseudoaneurysms have focused on open or endovascular parent artery sacrifice with or without revascularization [3, 4]. Coil or onyx embolization and use of stent grafts or covered stents have also been described in literature [5–8]. Recently, the use of flow-diverting stents in the management of traumatic pseudoaneurysms has been described [9]. We report a case of iatrogenic vertebral artery pseudoaneurysm that was successfully treated using the Pipeline Embolization Device (PED) (Ev3 Neurovascular, Irvine, CA), thus obtaining vascular reconstruction and excluding the aneurysm from circulation without compromising blood flow through the parent artery.

## 2. Case Report

**Figure 1:**
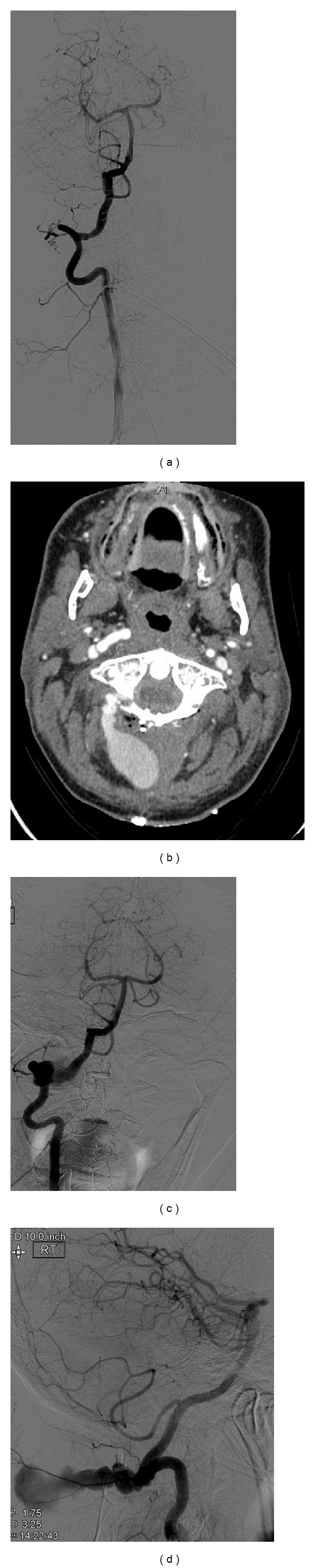
(a) shows normal filling of the right vertebral artery immediately following intraoperative injury: a small irregularity in the wall can be seen. (b) (CT angiogram) and (c) and (d) (digital subtraction angiogram) show a large saccular pseudoaneurysm arising from the V3 segment of the vertebral artery. The imaging was performed on the third postoperative day.

A 47-year-old man presented with pseudofusion following an old odontoid fracture. The patient was planned for posterior C1-C2 fixation using screws and rods. During dissection around the C1 lateral mass, on the right side, sudden brisk arterial bleeding was encountered. Immediate packing was done and hemostasis achieved. Vertebral angiography revealed patent VAs without any evidence of lumen compromise or active extravasation of contrast. A small irregularity on the wall was identified as the possible area of injury although no intimal flaps or flow limiting lesion was observed (Figure 1(a)). The patient underwent sublaminar wiring of C1 and C2 and was taken to the intensive care unit in a stable condition. Two days after the surgery, he developed a pulsatile swelling at the operative site. CT angiogram revealed a 5.5 × 2.0 cm pseudoaneurysm arising from the right vertebral artery (V3 segment), just superior to the posterior arch of C1 (Figure 1(b)). The patient was counseled regarding open and endovascular treatment options including parent artery sacrifice and vessel lumen reconstruction. Parent artery sacrifice was strongly considered; however, after discussion with the patient an attempt to preserve blood flow through the vertebral artery while excluding the aneurysm was decided upon.

## 3. Endovascular Treatment

**Figure 2:**
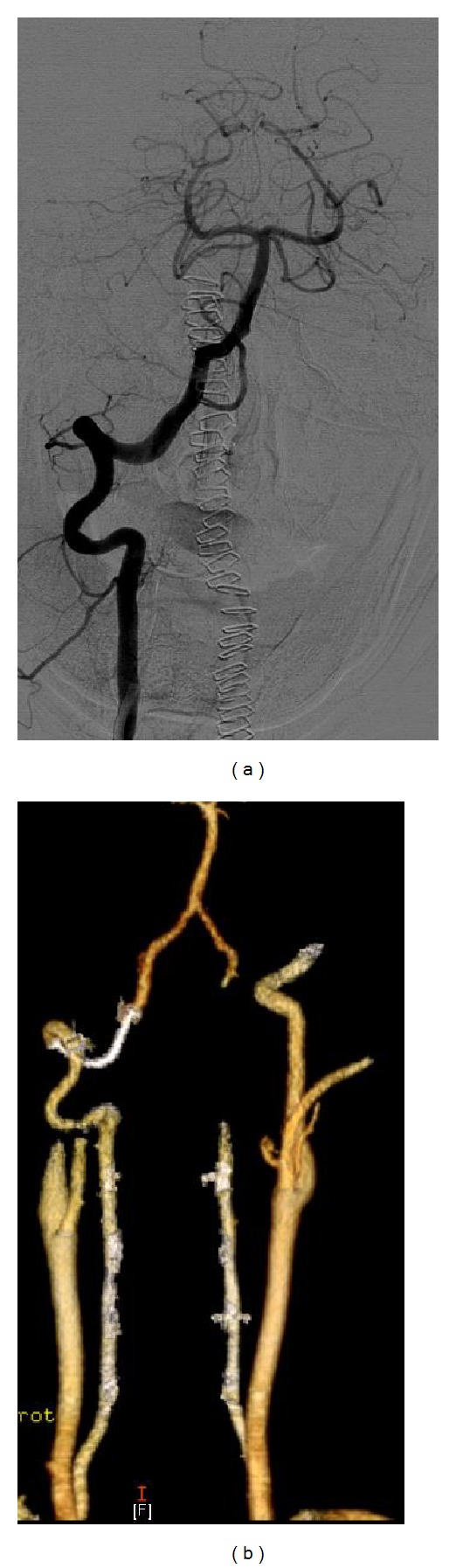
(a) shows right vertebral injection image immediately after deployment of the Pipeline Embolization Device. There is nonfilling of the pseudoaneurysm while the vertebral artery fills normally. (b) shows stable complete pseudoaneurysm occlusion and patent right vertebral artery in the CT angiogram performed at 10-month follow-up.

The patient received a dose of 600 mg Plavix PO 1 day and 3 hrs prior to the procedure. The procedure was performed using 1% lidocaine as local anesthetic and standard Seldinger technique to access the left femoral artery and to place 5-French sheath. A 5F Envoy catheter was advanced over a 0.035 guidewire and placed at the distal cervical segment of the right vertebral artery. A DSA run showed the pseudoaneurysm arising from the V3 segment and measuring approximately 30 mm long × 10 mm high with a 6 mm neck (Figures 1(c) and 1(d)). A Marksman microcatheter (Ev3 Neurovascular, Irvine, CA) was then advanced over a 0.012 microguidewire and selectively placed distal to the neck of the pseudoaneurysm. A 5 × 18 mm Pipeline Embolization Device was deployed over the neck of the pseudoaneurysm obtaining immediate complete occlusion of the pseudoaneurysm (Figure 2(a)). The sheath was removed and a 6-French Angio-Seal is used as a closure device. The patient remained on Plavix 75 mg PO daily for 6 months and was then switched to aspirin 81 mg indefinitely. The patient did well and is asymptomatic at the last follow-up 10 months after the procedure (Figure 2(b)).

## 4. Discussion

Pseudoaneurysms typically lack a true wall and are covered by a friable layer of connective tissue. Two types of iatrogenic pseudoaneurysms are reported, saccular and fusiform. Saccular pseudoaneurysms result from a focal, transmural injury to the vessel wall whereas fusiform pseudoaneurysms occur following dissection of the vessel resulting in thinning of adventitia and dilatation of the vessel [10]. Because of the lack of true neck, surgical or endovascular treatments have traditionally focused on parent vessel sacrifice with or without revascularization. Recently, management of these lesions has shifted from vessel destructive to vessel reconstructive strategies and various endovascular techniques are being employed to exclude the aneurysm from circulation while maintaining vessel patency [5, 6, 8].

The micropore flow-diverting stents are the most recent development in endovascular therapy of intracranial aneurysms. They were designed to obtain a greater change in aneurysm hemodynamics resulting in a diversion of flow within the stent. The SILK (SFD, Balt Extrusion, Montmorency, France) and Pipeline Embolization Device (PED, Covidien Vascular Therapies, Mansfield, MA, USA) promote flow diversion while preserving patency of branch vessels and perforating arteries. These flow-diverting devices have a larger metallic surface, thus having a greater potential to induce thrombosis within the aneurysm and endothelization of the neck of the aneurysm. In a recent systematic review, PED achieved 82.9% aneurysm obliteration at 6 months. The incidences of periprocedural complications and mortality were 6.3% and 1.5%, respectively [11].

Flow diversion has also been used in the management of dissecting carotid and vertebral artery aneurysms with occlusion rates up to 87.5% [12]. Amenta et al. reported a case of traumatic carotid pseudoaneurysm successfully treated using the PED [9]. Although flow-diversion stents have not been used earlier for treatment of traumatic vertebral pseudoaneurysms, we believe that they can be used to exclude the pseudoaneurysm while maintaining vessel patency. A caveat to the statement is that there is a higher possibility of successful outcome if the acute bleeding from the pseudoaneurysm has ceased at the time of treatment since the success of flow diversion is dependent on lack of significant pressure gradient across the wall of the pseudoaneurysm. Hence, use of a PED in actively bleeding traumatic pseudoaneurysms is likely not useful and potentially contraindicated. Since the flow-diversion technique causes thrombosis within the aneurysm over a period of time, there remains a risk of rupture of the pseudoaneurysm in the immediate postprocedure period. Further investigation into the blood flow hemodynamics of pseudoaneurysms following PED treatment is warranted to be able to treat these lesions more effectively and safely.

## 5. Conclusion

Iatrogenic pseudoaneurysms are rare, challenging lesions. The Pipeline Embolization Device, which has been effectively used in the management of complex aneurysms, may effectively be used in the management of these lesions while maintaining vessel lumen patency.
